# Survival of the first rather than the fittest in a *Shewanella* electrode biofilm

**DOI:** 10.1038/s42003-021-02040-1

**Published:** 2021-05-06

**Authors:** Eric D. Kees, Caleb E. Levar, Stephen P. Miller, Daniel R. Bond, Jeffrey A. Gralnick, Antony M. Dean

**Affiliations:** 1 BioTechnology Institute, University of Minnesota, St Paul, MN USA; 2 Department of Plant and Microbial Biology, University of Minnesota, St Paul, MN USA; 3 Department of Ecology, Evolution and Behavior, University of Minnesota, St Paul, MN USA

**Keywords:** Bacteriology, Experimental evolution

## Abstract

For natural selection to operate there must exist heritable variation among individuals that affects their survival and reproduction. Among free-living microbes, where differences in growth rates largely define selection intensities, competitive exclusion is common. However, among surface attached communities, these dynamics become less predictable. If extreme circumstances were to dictate that a surface population is immortal and all offspring must emigrate, the offspring would be unable to contribute to the composition of the population. Meanwhile, the immortals, regardless of reproductive capacity, would remain unchanged in relative abundance. The normal cycle of birth, death, and competitive exclusion would be broken. We tested whether conditions required to set up this idealized scenario can be approximated in a microbial biofilm. Using two differentially-reproducing strains of *Shewanella oneidensis* grown on an anode as the sole terminal electron acceptor – a system in which metabolism is obligately tied to surface attachment – we found that selection against a slow-growing competitor is drastically reduced. This work furthers understanding of natural selection dynamics in sessile microbial communities, and provides a framework for designing stable microbial communities for industrial and experimental applications.

## Abstract (web summary)

Eric Kees et al. explore whether ecological competition differs between surface-attached biofilms and planktonic culture using two differentially-reproducing strains of *Shewanella oneidensis*. They observed that, regardless of different growth rates, the first bacterial species to colonize a surface would persist and would not be excluded by a faster growing competitor.

## Introduction

Natural selection, the differential survival, and reproduction of competing genotypes, is a cornerstone of modern evolutionary theory and the only mechanism known to produce organismal adaptations^1^. Adaptive evolution requires competitive exclusion, the displacement of a competitor by one better suited to its environment^2^. Although this competitive exclusion principle is readily demonstrated in microbial competition experiments^3–5^, there exist many exceptions to the rule^6–8^, all of which involve reduced niche overlap. For example, competitors might be limited by different resources^9^, favored at different times^10,11^, or in different places^12,13^. For each exception, reduced niche overlap promotes a diversity of coexisting organisms, while complete niche overlap excludes all but the fittest.

Here, we explore a set of conditions presumed to produce complete niche overlap, but with minimal competitive interaction. Under an idealized scenario (described and modeled in detail in Supplementary Note), a colonizing surface-attached population would experience no death or detachment and would thus not be replaced or overtaken by its progeny or competing cells. Under these conditions, the first organisms to colonize a surface would persist regardless of their capacity for growth, allowing for the initial diversity of a founding population to be preserved. We ask whether conditions approaching this extreme are achievable in laboratory culture, and whether surface attachment—under benign conditions (i.e., without substantial stress or externally imposed death or surface abrasion) and absent cooperative or antagonistic interactions—is sufficient to stabilize relative abundance of two strains using the same space and nutrients, but with differing growth rates.

A microbial biofilm, specifically one attached to a non-degrading surface under constant medium supply, provides a practical experimental system in which to test surface competition in an actively dividing population^14,15^, without introducing environmental fluctuations that may drive selection. *Shewanella oneidensis*, a genetically tractable facultative anaerobe capable of growth using a variety of soluble and insoluble terminal electron acceptors^16,17^, including graphite electrodes poised at anodic potentials^18,19^, is an attractive biological platform for surface competition experiments. When grown on graphite anodes under strict anaerobic conditions, *S. oneidensis* forms stable biofilms that rarely become multilayered^19^ and do not degrade the surface substrate, simplifying monitoring of strain abundances over long-term competitions. We chose two strains of *S. oneidensis*, each derived from the same lab strain, MR-1, to serve as competitors in this study. Strain ∆*llpR* has a deletion in the l-lactate positive regulator gene *llpR* which results in impaired expression of the l-lactate dehydrogenase and slowed growth on l-lactate^20^. The faster growing strain (with growth rate nearing wild-type), MR-1 + *gfp*^21^, expresses green fluorescent protein from a genomic copy of gfpmut3*^22^ which allows for independent strain quantitation and identification in mixed populations by flow cytometry, dilution plate counting, and confocal microscopy.

**Fig. 1:**
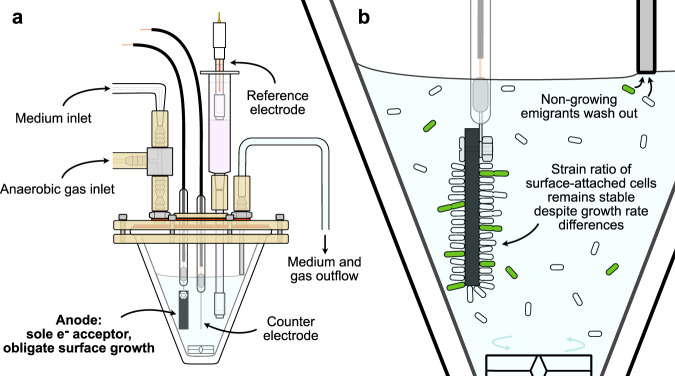
Experimental platform. **a** Anaerobic three-electrode bioreactor. Fresh sterile anaerobic medium is pumped at a constant rate into a growth chamber under positive pressure from a flow of sterile humidified argon. Both spent medium and argon exit through an air-locked outlet. Cells are grown on a graphite working electrode poised at anodic potentials with a platinum counter electrode and a Ag/AgCl reference electrode to control the voltage. A magnetic stir bar mixes the planktonic phase. **b** Only cells attached to the anode can grow. Hence, the strain ratio in the planktonic phase equals the ratio on the anode times the relative growth rate (see Note in Supplementary Information).

To further simplify testing of our central hypothesis that surface attachment can minimize competition, we imposed a requirement that only surface-attached cells may be permitted to grow, allowing for quantitation of the surface population by counting planktonic emigrants. Unless specifically engineered to ferment pyruvate^23^, *S. oneidensis* is unable to metabolize and grow in the absence of a terminal electron acceptor^16,24^, therefore no growth occurs in the planktonic phase when a graphite anode is provided as the sole electron acceptor. While 250 nM riboflavin was added to continuously fed cultures to mitigate washout of flavins typically secreted by *S. oneidensis* under anaerobic conditions^25^, this concentration is not sufficient to facilitate significant reduction of the electrode by planktonic cells^26^. Moreover, a mutation causing a flavin secretion defect does not result in a significantly different amount planktonic cells in both batch- and continuously fed anaerobic electrode biofilm cultures than wild-type, further suggesting that shuttling to electrodes via physiological concentrations of flavins does not support planktonic growth^27^. Motility, and the possibility of electrode recolonization by planktonic cells^28^, is also limited due to the lack of soluble terminal electron acceptors as taxis requires the generation of proton motive force. Thus, since planktonic emigrants from the electrode surface will be non-growing, our experimental system, a continuously fed anaerobic three-electrode bioreactor (Fig. 1a) enables competition on the anodes to be monitored by counting the planktonic population (Fig. 1b).

## Results

**Fig. 2:**
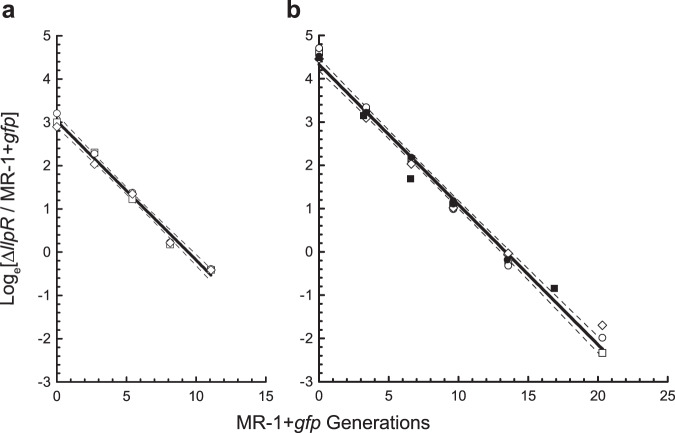
Planktonic competition. Anaerobic competition for lactate with fumarate as the terminal electron acceptor. The slopes of the solid lines (selection coefficients per MR-1 + *gfp* generation estimated by linear regression) reveal strong selection against strain ∆*llpR* when placed in competition with strain MR-1 + *gfp*. **a** Three replicate competitions in planktonic batch growth with an overall selection coefficient of −0.321 ± 0.010 gen^−1^ (relative growth rate *w* = 0.536 ± 0.014). **b** Five replicate competitions across two experiments in bioreactors with electrodes unpoised; overall selection coefficient of −0.324± 0.007 gen^−1^ (relative growth rate *w* = 0.533 ± 0.010). Different symbols (white or black diamonds, circles, and squares) represent replicate competitions. Dashed lines indicate 95% confidence bands derived from linear regression.

We first tested whether the two strains of *S. oneidensis* selected for competition conform as expected to the competitive exclusion principle when grown together in planktonic cultures. As measured in anaerobic planktonic batch-fed cultures, with l-lactate as the carbon source and fumarate as the electron acceptor, strain ∆*llpR* grew approximately half as fast as MR-1 + *gfp* (specific growth rates are 0.329 ± 0.005 h^−1^ and 0.627 ± 0.009 h^−1^ respectively), in agreement with previous growth measurements of strain ∆*llpR*^20^, and MR-1 + *gfp*^21^. When placed under batch-fed conditions in competition, strain ∆*llpR* was rapidly overtaken by MR-1 + *gfp* (Fig. 2a). The observed selection coefficient (the slope of the line of the log ratio of strains plotted against time; see equation 5 in Supplementary Note) is = −0.291 ± 0.009 h^−1^, which is very similar to the predicted value of 0.329 −0.627 = −0.298 h^−1^, obtained as the difference between the growth rate of each strain in pure culture. The growth rate of ∆*llpR* relative to MR-1 + *gfp* (a measure of fitness) is *w* = 1 − 0.291/0.627 = 0.535 ± 0.011. Similar to batch planktonic competitions, strain ∆*llpR* was also quickly outcompeted by MR-1 + *gfp* when grown planktonically in unpoised three-electrode bioreactors with l-lactate and fumarate given upon inoculation and replenished by medium feed (Fig. 2b). The selection coefficient *s* = −0.243 ± 0.005 h^−1^ is smaller than that seen in the batch culture competitions, but both strains grew slower and thus the relative growth rate of ∆*llpR*, *w* = 1 – 0.243/0.520 = 0.533 ± 0.011, was unchanged. Together, these control experiments establish that competitions between strains ∆*llpR* and MR-1 + *gfp* conform to the competitive exclusion principle during planktonic growth.

**Fig. 3:**
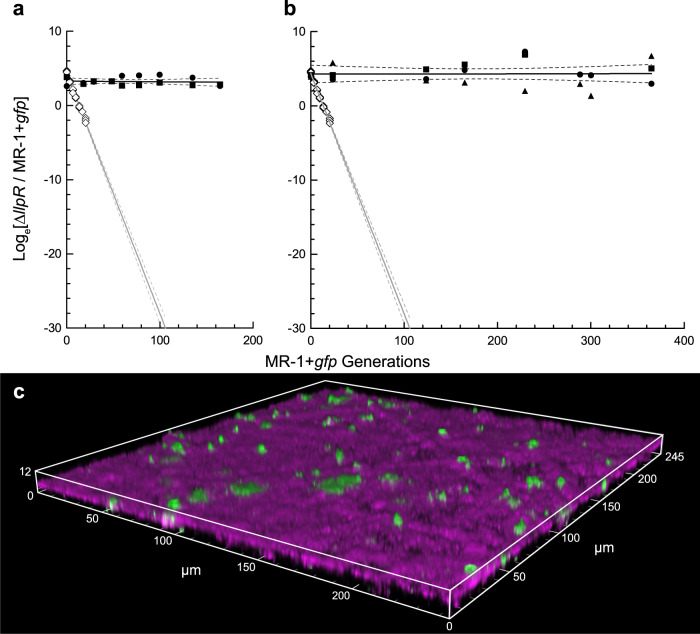
Biofilm competition. Anaerobic competition for lactate in bioreactors with electrodes poised (black circles, squares, and diamond, along with black lines). **a** Two replicate competitions conducted over 671 h (~165 MR-1 + *gfp* divisions) with abundances of each strain measured by flow cytometry. **b** Three replicate competitions conducted for 1488 h (~365 MR-1 + *gfp* divisions) and measured by dilution plate counting. For comparison, gray lines extrapolate the planktonic selection in bioreactors with unpoised electrodes (white diamonds). **c** Confocal microscopy volume projection of a mixed-culture biofilm at 929 h confirms that MR-1 + *gfp* cells (green) still sparsely populate the biofilm which remains dominated by ∆*llpR* cells (magenta). Symbols indicate experimental replicates (i.e., one symbol for each bioreactor), solid and dashed lines indicate linear regression and associated 95% confidence bands, respectively.

Next, we tested surface-attached competition between strains ∆*llpR* and MR-1 + *gfp* in customized, three-electrode anaerobic bioreactors which were constantly replenished with medium (Fig. 1a), and in which only cells attached to anodes can grow due to a lack of soluble terminal electron acceptor (Fig. 1b). In marked contrast to the planktonic competitions, the ratio of strains ∆*llpR* and MR-1 + *gfp* on poised graphite anodes, as reflected by their planktonic relative abundance, remained unchanged over the course of the experiments (Fig. 3). The strongest estimated selection coefficient for ∆*llpR* among two separate anodic competition experiments was −0.00082 ± 0.00246 gen^−1^ estimated for 671 h of growth (Fig. 3a), which is not significantly different from zero (*P* = 0.7428). Such selection indicates that many generations would be required to achieve sweeping changes in relative strain abundances. For example, if the MR-1 + *gfp* strain were inoculated against the ∆*llpR* strain at a starting abundance of 1 cell per 1 million (Log_e_ × 1/10^6^ = −13.82), it would be expected to cross a threshold of 50% (Log_e_ × 1/1 = 0) after ~8 years of continuous culture (~16,800 generations). Alternatively, if selection had followed the selection coefficients seen in planktonic bioreactor growth (Fig. 2b), after 365 MR-1 + *gfp* generations, strain ∆*llpR* would have been expected to decline from its initial abundance of ~0.98 to 0.98 × 0.533^365^ = 1.765/10^100^. Instead, bioreactors operated for 365 generations or 1488 h provided no evidence of selection for ∆*llpR* (+0.0001517 ± 0.002633 gen^−1^) when assayed for colony forming units (Fig. 3b). End-point confocal microscopy showed that biofilm structure remained relatively thin over the course of the experiments, with both strains growing as single cells or in microcolonies with minimal structure or lateral spread (Fig. 3c). These results are consistent with the hypothesis that surface attachment stabilizes strain ratios in a population competing for the same space and nutrients. However, these results alone are not sufficient to conclude that conditions in our experimental system approached the idealized scenario, in which parents divide but are not replaced by progeny. First, we must address several alternative hypotheses to explain the stable planktonic relative abundances observed in our bioreactors: (1) Surface-attached cells did not grow, (2) surface-attached growth rates are equal, (3) planktonic growth occurred for both strains at equal rates, (4) the slower growing strain had an intrinsically higher colonization rate, and (5) the slower growing strain was given a colonization advantage by its high initial seeding ratio relative to the faster growing strain.

The first alternative hypothesis, that neither strain divides in the bioreactors —and thus measured planktonic strain ratios reflected cells sloughed from a non-growing biofilm population—is readily dismissed. Given a dilution rate of 0.1 h^−1^ (approximate to that in our surface competitions), a non-growing population should decline by a factor of *e*^−0.1 × 1488^ = 2.4 × 10^−65^. Thus, absent growth, no measurable population of cells should have remained in the bioreactors. Yet total planktonic populations largely plateaued by 300 h and did not change significantly over the remainder of the experiments (Fig. S1a) except when perturbed by addition and subsequent removal of a soluble electron acceptor (Fig. S1b).

**Fig. 4:**
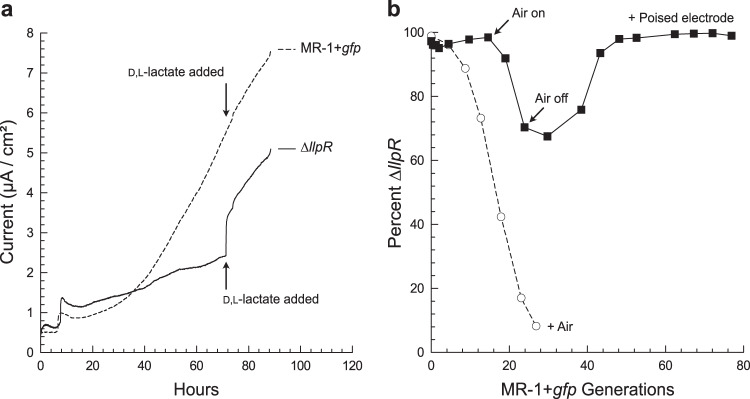
Alternative hypothesis testing. **a** The carbon source determines the metabolic rate of strain ∆*llpR* (solid line). Adding d,l-lactate provides a second carbon source, d-lactate, that is utilized preferentially and equally by both strains. The immediate increase in current from strain ∆*llpR* (but not from strain MR-1 + *gfp*, represented by a dashed line) demonstrates that it is limited by its capacity to metabolize l-lactate during growth on electrodes. Data shown is representative from *N* = 2 experimental replicates. **b** Letting air into an anaerobic bioreactor (black squares and solid line) provides planktonic cells with O_2_ as a terminal electron acceptor. The ensuing competition causes a decline in the relative abundance of strain ∆*llpR*. Once anaerobic conditions have been re-established, strain ∆*llpR* returns to its original frequency, reflecting migration from a biofilm that is resilient to such perturbations. For comparison, strain ∆*llpR* is at a strong disadvantage during competition in an aerobic planktonic culture (white circles and dashed line). Data shown represent a single experiment.

The second alternative hypothesis states that while planktonic growth rates between strains are different, surface-attached growth rates are equal. The primary mechanism through which this hypothesis could explain stabilized strain ratios in our biofilm competitions is, if relative anodic growth rates were limited by the rate at which the anode could be respired, rather than controlled by the capacity to metabolize l-lactate. We measured anodic respiration rates (electrical current received by the anode) upon the addition of d-lactate, a carbon source that *S. oneidensis* preferentially utilizes over l-lactate and which both strains metabolize equally well^20^. Adding d,l-lactate to pure cultures grown on anodes with l-lactate as the carbon source causes an immediate increase in current from strain ∆*llpR*, but not from strain MR-1 + *gfp* (Fig. 4a). The increased current from strain ∆*llpR* demonstrates that, relative to strain MR-1 + *gfp*, its metabolism on anodes (and, by inference, its growth rate) is limited by its capacity to metabolize l-lactate. To account for other unforeseen mechanisms by which anodic growth rates might be equal for both strains, we further estimated growth rates by proxy of dilution rate and the densities of cells in both the planktonic phase and on the electrodes (see equations 10 and 11 in Supplementary Note). Growth rates estimated for each strain were much slower on the anodes than in batch-fed planktonic cultures (0.083 ± 0.005 h^−1^ and 0.170 ± 0.009 h^−1^ for ∆*llpR* and MR-1 + *gfp*, respectively; mean and SEM for *N* = 32 growth rate calculations). Nevertheless, the estimated growth rate of *∆llpR* relative to MR-1 + *gfp* on anodes (*w* = 0.083/0.170 = 0.488 ± 0.054; *p* < 0.0001 by Welch’s *t*-test; Fig. S2a) was similar to that in planktonic cultures (*w* = 0.53 ± 0.01). Since respiration rate is a suitable proxy for growth rates, we collected a second estimation of relative growth rate, by measuring respiration rates of each strain normalized to biomass. As might be expected of cells that grow at half the rate, *∆llpR* cells generated half the current (µA/µg protein) of MR-1 + *gfp* cells (0.534 ± 0.077; *p* = 0.0175 by paired *t*-test; *N* = 3 replicate experiments, each with two replicate samples per strain; Fig. S2b). From the results of these three experimental methods, we conclude that the relative growth rate is unaffected by attachment.

The third alternative hypothesis to explain the stable strain ratios observed in biofilm competitions, posits the presence of a limited concentration of terminal electron acceptors that supports planktonic growth by both strains, but at the same rate. This might occur in our bioreactor through diffusion of air into the medium lines and/or electron shuttling to and from the anode via soluble intermediates^29,30^. To test this hypothesis, we introduced a soluble electron acceptor into the medium and then monitored perturbed abundance of strains during outgrowth of the planktonic population. Assuming planktonic strain ratios were driven by biofilm emigration, we expected perturbed relative abundances to return to a prior state upon removal of the soluble electron acceptor, as new emigrants would replace the outgrown planktonic population as it washed out. Alternatively, if planktonic strain ratios were driven by slow equal planktonic growth, we would expect perturbed relative abundances to persist upon removal of the soluble electron acceptor, as planktonic growth would continue but at a slower rate. When air was introduced into an anaerobic bioreactor to provide planktonic cells with O_2_ as a soluble terminal electron acceptor, the planktonic relative abundance of strain ∆*llpR* declined (Fig. 4b) as expected. However, once the air ingress was eliminated and an anoxic environment was restored, strain ∆*llpR* returned to its initial abundance (~98%). These results do not support the alternative hypothesis that stable planktonic strain ratios measured in the biofilm competition experiments were driven by acceptor-limited planktonic growth. Rather, the return of strain ∆*llpR* to its original abundance suggests not only that electrode-attached cells primarily drove planktonic strain ratios in biofilm competitions, but also that surface-attached population ratios were resistant to recolonization by an actively dividing planktonic population. A similar outcome occurred when bioreactors were spiked with another soluble electron acceptor, fumarate (Fig. S3).

The fourth alternative hypothesis states that the competed strains grew at different rates and replaced the parental population, but had surface colonization rate differences that compensated for growth rate differences and stabilized observed strain ratios. Although intrinsic colonization rate differences between the two competed strain are unlikely—genetically they differ only by addition or deletion of two genes unrelated to attachment—we sought to address them. We observed considerable variation in electrode colonization estimates among experimental batches by measures of surface-attached cell counting and biofilm protein quantitation. For example, two pooled experiments measuring cells per unit area of electrode via confocal microscopy showed significantly higher colonization by ∆*llpR* than by MR-1 + *gfp* (∆*llpR* = 3.2 × 10^7^ ± 5.1 × 10^6^ cells/electrode; MR-1 + *gfp* = 1.4 × 10^7^ ± 1.5 × 10^6^ cells/electrode; *p* = 00018; *t*-test with Welch’s correction for unequal variance; total *N* electrodes per strain = 4). Biofilm protein measurements also displayed batch to batch variation, but yielded colonization estimates that contradict those from cell counting, albeit without statistical significance, with insignificantly higher levels of total biofilm protein measured for the MR-1 + *gfp* strain across three experiments (∆*llpR* = 135 ± 29 μg protein/electrode; MR-1 + *gfp* = 185 ± 12 μg protein/electrode; *p* = 0.1253 by paired *t*-test; *N* = 3 electrode pairs, with two averaged replicates for each strain per experiment). Considering contradicting results depending on measurement method, along with large batch-to-batch variation, this alternative hypothesis remains neither rejected nor supported by our experiments. However, given the genetic pedigree of each strain, it is more likely that differences in bioreactor and experiment preparation—such as electrode surface preparation, particulate matter in medium, size and growth phase of inoculum—explain inconsistent colonization of each strain among the experiments conducted, rather than potential intrinsic colonization rate differences between strains.

Similar to the fourth alternative hypothesis, a fifth and final alternative hypothesis states that the initial seeding ratio of 9:1 ∆*llpR* to MR-1 + *gfp* in surface-attached competition experiments gave a colonization advantage to ∆*llpR* that compensated for growth rate differences. The seeding ratio was chosen primarily to allow for a greater number of generations to measure relative abundance changes by flow cytometery and to give the fast-growing variant an opportunity to overtake the slow-growing variant. Thus, real, sweeping changes in MR-1 + *gfp* abundance over multiple time points were easier to detect when it was placed at a low initial abundance. However, the potential that a 9:1 ratio led to favorable recolonization by strain ∆*llpR* in the event of parental death was considered. Under this fifth alternative hypothesis, a colonization advantage for either strain at any given time would be directly proportional to its relative abundance, and any deviation from a strain ratio that balances with intrinsic growth rate differences would result in runaway selection in favor of one strain. In bioreactors that varied in both initial starting ratios across replicates and measured relative abundances over time, we did not observe sustained runaway selection (see experimental replicates in Fig. 3), but instead observed overall insignificant changes in strain ratios in each replicate. When considering variation among replicates, neither alternative hypothesis stating that colonization differences compensate for growth rate differences appear to explain the stable population composition observed in our surface-attached competition experiments.

## Discussion

We conclude that conditions approaching an idealized scenario allowing for prolonged stabilization of population composition were achieved in our experiments via surface attachment: an actively dividing founding population persisted with minimal apparent interaction between potential competitors. As evidenced by observed near-neutrality over 365 generations of growth, we expect that *S. oneidensis* death rates were sufficiently small to have not significantly driven competition. Intriguingly, laboratory grown *Escherichia coli*^31,32^ and *Saccharomyces pombe*^33^ cells are effectively immortal unless exposed to extrinsic stresses. This suggests that some microbial cells might generally have very low intrinsic death rates under benign laboratory conditions such as those used in this experiment. The observation that replacement of parents by progeny does not appear to occur at appreciable levels in our system establishes a scenario in which selection against an entrenched viable population, whether via recolonization or invasion by better suited competitors, is prevented due to a lack of occupiable space. Like selection, random genetic drift (accumulated random changes in competitor frequencies) is also expected to have been minimized due to a lack of occupiable space and thus a limited capacity for progeny to shape the genetic makeup of the surface population. Of the three evolutionary forces at play, only mutation is left. However, mutation is too weak to be an effective evolutionary force^34^. Assuming a loss of function mutation rate of 10^−6^ per gene per generation it would take 4.6 × 10^6^ generations (or 4.6 × 10^6^/0.170/24/365 = 3089 years) for an allele to mutate from a frequency of 0.99 to 0.01. Evolution has, for all practical intents and purposes, either ceased or slowed to immeasurable rates in these anode biofilm competition experiments over the timescales presented here.

Previous research exploring biofilm diversity has demonstrated that it is highly dependent on structure^35,36^, although for very different reasons than we explored here. Particularly, social interactions and spatial heterogeneity in resources promote niche diversity within biofilm communities^37–40^. Furthermore, spatial structure can promote yield maximizing metabolic strategies over growth maximizing strategies^41^, and negative social interactions promote segregation between strains, which in turn can promote coexistence^42–45^. Although we demonstrate that surface attachment itself can promote diversity by minimizing interactions among competitors and thus limiting the potential for selective sweeps by more fit individuals, the question remains of whether this scenario can be observed or achieved in other natural or engineered systems. Evolution over similar timescales as tested in our experiments, has been observed in other biofilms. For example, at least three distinct morphotypes of *Burkholderia* developed in long-term cultures propagated using a serial transfer regime in which bacteria attached to polystyrene beads in the current tube must disperse to colonize fresh beads in the next tube^35^. In contrast to our methods, these experiments imposed selection via serial transfer: (1) more efficient colonizers are favored at each transfer and (2) faster growing surface-attached bacteria, being more abundant in the planktonic phase, will differentially colonize the fresh surfaces at each transfer. Other studies demonstrate evolved diversification of a biofilm population (e.g., natural selection driving changes in community composition)^46,47^. In these studies, strains with increased fitness rapidly overtake the system, in contrast to the near-neutral selection against a slower growing strain observed in our poised electrode system.

Since the idealized selection-less scenario we present is predicated on a condition of full maintenance of a parental population, any biofilm in which death or removal by predation, stress, abrasion, or invasion is present is unlikely to approach this extreme for sustained periods. Testing whether surface attachment stabilizes relative strain abundances in other biofilms is further complicated by myriad potential social interactions, whether cooperative or antagonistic. Thus we expect that the prolonged stability we observed in *Shewanella* biofilms on an anode surface is fairly unique compared to other biofilms, at least when taken in whole. Transient periods in natural biofilms in which benign conditions may promote attachment-mediated resistance of a growing population to recolonization by progeny or outside competitors are possible. We further anticipate other engineered biofilms approaching the extreme of minimized competitive selection on a founding population. This type of persistence in engineered *Shewanella* biofilms could be of particular interest when considering the genetic tractability of *S. oneidensis* and the ability to link the production of a desired product, such as acetoin, to electrode respiration^48–50^. Such processes can be hindered by the emergence of cheaters, populations no longer contributing to the end product formation. Using electroactive biofilms for these types of processes may have the potential to reduce the emergence of these cheating populations, resulting in an overall more stable biofilm for production of industrial products. Moreover, this work suggests that the initial colonization of electrodes in microbial fuel cells by mixed communities (e.g., wastewater) of high and low current producing organisms may limit the overall performance of the system because poor performing residents may not readily be displaced by strong performers. A strategy to mitigate this problem would be to seed electrodes with strong current producers at system startup.

Further research will be required to determine what conclusions from this study could be applicable to other microorganisms, to more complex communities, or to systems where the surface is not metabolically linked to the growth of the organism. For example, one can envision wall growth in a chemostat system with large surface area to volume ratios and flow rates sufficient to limit planktonic growth may also limit natural selection. This type of design could replace large industrial batch fermenters with smaller, more economical, and potentially more stable, continuous culture bioreactors. However, such a system may also be prone to evolutionary forces not present when using a metabolically linked surface. Within the theoretical framework presented here the immensely powerful force of natural selection is negated only under an idealized condition in which a population of immortals produces offspring that must emigrate. While we entertain the notion that conditions approaching this ideal may arise naturally in some biofilms, our conclusions are constrained to the experimental system tested here, in which attachment and metabolism are inexorably linked. Under these specific circumstances we observed a lack of selection, a system favoring the first rather than the fittest.

## Methods

### Strains

Both experimental strains are derived from wild-type *S. oneidensis* MR-1^16,17^ lab strain JG273, and strain Δ*llpR* is described previously^20^. Strain MR-1 + *gfp*^21^ was constructed by insertion of gfpmut3*^22^, under control of the constitutive promoter A1/04/03^51^, into the neutral attTn7 insertion site downstream of gene *glmS* in MR-1^52,53^. A modified double homologous recombination method^54^ was used to target the gene insertion.

### Media

*Shewanella* Basal Medium (SBM) consisted of 0.225 g K_2_HPO_4_, 0.225 g KH_2_PO_4_, 0.46 g NaCl, 0.225 g (NH_4_)SO_4_, and 0.117 g MgSO_4_·7H_2_O per liter, adjusted to pH 7.2 and buffered with 10 mM HEPES^55^. Medium was supplemented with 0.05% casamino acids (Fisher), and 5 mL/L each of vitamins and mineral mix described previously^56^. In anaerobic cultures, lactate and fumarate were used, respectively, as carbon source and terminal electron acceptor, and their concentrations are described where noted.

Unless otherwise noted, inocula for all cultures were routinely prepared by growing and isolating colonies on lysogeny broth (LB) 1.5% agar plates, transferring single colonies into LB and growing for 8 h with aeration, then transferring 10 μL into SBM containing 20 mM lactate and growing aerobically overnight. Media reservoirs for all continuous-flow experiments were supplemented with 250 nM riboflavin to correct for washout of flavin electron shuttles^25^.

### Anaerobic planktonic competitions

Strains MR-1 + *gfp* and Δ*llpR* were grown together in sealed, nitrogen-flushed anaerobic tubes containing SBM supplemented with 20 mM l-lactate and 40 mM fumarate. Initial inocula were set at a ratio of 97:3 strain Δ*llpR*: MR-1 + *gfp*, and tubes were inoculated at a starting density of 0.010 OD_600_. Exponential-phase (OD_600_ = 0.200) cultures were transferred to fresh anaerobic medium via syringe to maintain exponential growth. For flow cytometry, samples taken at each time point were exposed to air for 10–20 min to facilitate GFP folding and diluted to 10^5^–10^6^ cells/mL in 0.2 µm-filtered SBM to determine percent GFP and non-GFP expressing cell abundance by flow cytometry. For plate counts, samples were diluted in SBM, 100 µL was spread on two separate LB-1.5% agar plates, which were incubated first for colony growth at 30 °C for 18 h, then for maximal GFP fluorescence at 4 °C for 4–24 h. Colonies formed by strains MR-1 + *gfp* and Δ*llpR* were differentiated and counted under UV light.

### Three-electrode bioreactors

The three-electrode chambers consist of a 3 cm^2^ polished graphite electrode, an Ag/AgCl reference electrode, and a Pt counter electrode within a polyether ether ketone (PEEK) topped 35 mL glass cone chamber, with inlet, outlet, and sampling ports. At the inlet port, media was fed by peristaltic pump (Ismatec IPC) through Pharmed 0.25 mm ID BPT tubing (Cole Parmer), and the headspace was continuously flushed with argon. Argon gas was scrubbed of oxygen via a heated copper catalyst, and was humidified at the inlet port by bubbling gas through vials with sterile milliQ water. At the outlet port, a small custom stainless-steel pickup tube was fitted to set the average medium volume at 13.3 ± 0.3 mL and allow for a sizable pressurized headspace. To minimize oxygen permeation into medium feed lines, stainless steel or glass fittings were used where possible. Redox potential was set at 240 mV vs SHE using a Bio-Logic SAS model MPG2 potentiostat, and EC-Lab software. Electrode respiration rate was monitored as an average taken over 2 min and expressed as the current (µA) per cm^2^ of electrode surface.

### Planktonic competition in bioreactors

Filtered SBM medium containing 2 mM l-lactate and 4 mM fumarate was fed through unpoised three-electrode chambers at an average dilution rate of 0.185 ± 0.005 h^−1^. Chambers were inoculated with 200 μL of a 95:5 mix of strain Δ*llpR*: MR-1 + *gfp*. One microliter samples were taken at routine time points and diluted fourfold in filtered SBM for flow cytometry measurements.

### Competition in anode-attached biofilms

To establish mixed-culture biofilms, 100 μL overnight LB monoculture of each strain was added to individual anaerobic tubes containing 10 mL SBM (30 mM d,l-lactate and 40 mM fumarate) and grown at 30 °C until mid-exponential phase. 1.1 mL of anaerobic mid-exponential MR-1 + *gfp* culture was added to 10 mL anaerobic ∆*llpR* culture to make an approximate 1:9 mix for inoculation. One microliter of the inoculum was then added to each of three bioreactor chambers containing filtered SBM with 30 mM d,l-lactate and 40 mM fumarate. A control bioreactor containing strain MR-1 + *gfp* alone was prepared similarly. Working electrodes were poised at 242 mV vs SHE and respiration rate was measured by chronoamperometry. When the respiration rate plateaued at 147 h, filtered SBM containing 2 mM l-lactate was fed into the chambers by peristaltic pump at a rate of 1.35 mL/h. Beginning 42 h after starting continuous media feed (after ~56 mL or over 4 medium volume replacements had washed through reactors), 1–2 mL samples were taken at regular time points and measured undiluted by flow cytometry.

### Flow cytometry

All measurements were conducted on a FACSCalibur flow cytometer (Becton Dickson), equipped with 488 and 640 nm lasers, using the FL1 green detection channel through a 530/30 filter. Flow rates were set to 35 µL/min and samples were taken in 53 s passes. GFP and non-GFP expressing populations were gated based on control cultures containing 100% MR-1 + *gfp* strain, using commercial FlowJo software. The gating strategy used in this study has been described in detail previously^21^.

### Confocal microscopy

Graphite electrodes containing biofilms were harvested from bioreactor chambers and stained with 5 μM SYTO^®^ dye 41 (ThermoFisher) in filtered SBM. Following 30 min staining, biofilms were imaged on a Nikon A1 confocal microscope using GFP and SYTO41/DAPI channels.

### Electrode biofilm growth rate determinations

Three-electrode chambers containing filtered SBM with 30 mM d,l-lactate and 40 mM fumarate were inoculated separately with mid-exponential anaerobic cultures of strain MR-1 + *gfp* or ∆*llpR*. Two bioreactors were used for each strain to account for variation in electrode surface preparation. After 180 h when respiration rate had plateaued, filtered SBM containing 2 mM l-lactate and 250 nM riboflavin was fed at an average rate of 1.34 ± 0.08 mL/h. Flow cytometry measurements on planktonic samples were taken at 279 and 300 h post-inoculation. Electrodes were harvested for staining and imaging at 301 h post-inoculation. To account for variation in electrode surface topology and biofilm density, two randomly subsampled regions of four representative 100x images (examples in Fig. S4) from each electrode were used to count cells within the FIJI distribution^57^ of ImageJ software^58^ using the Cell Counter plugin. Cell division rates were calculated from total number of attached cells per electrode (estimated from cell counts on confocal micrographs) divided by estimated planktonic cells/h removed from each bioreactor at each time point (derived from flow cytometry counts and instantaneous medium flow rate). Since no measurable planktonic division was occurring, dilution rate did not determine growth rate as in a chemostat. Thus, the total number of cells produced per hour from the electrode and lost to the planktonic phase must match the rate of cells lost from the planktonic phase at steady state due to continuous flow. *P* values are derived from student’s *t*-test with Welch’s correction to account for unequal variance.

### Total biofilm protein and normalized respiration rate measurements

Normalized current production for each strain was determined by dividing the total current produced by the total protein attached to the working electrode surface within a given bioreactor. Briefly, the carbon electrodes were carefully removed from bioreactors and placed in 2 mL microcentrifuge tubes containing SBM medium with no donor or acceptor. Tubes were inverted once to remove loosely attached cells from the electrode surface before electrodes were moved to a microcentrifuge tube containing 1 mL of 0.2 N NaOH. Cells still attached to the electrode surface were lysed by boiling the washed electrodes in 1 mL of 0.5 N NaOH for 10 min, agitating once after 5 min. The total protein attached to the electrodes was measured using bicinchoninic acid protein assay (BCA). Briefly electrodes were placed in 1 mL buffered medium lacking donor and acceptor and inverted one time to remove loosely attached cells. Electrodes were then placed in a 2 mL microcentrifuge tube containing 1 mL of 0.5 N NaOH and heated for 5 min at 95 °C for 5 min, mixing by vortex once for 2.5 min. Serial dilutions were then performed from the resulting suspension, and the Pierce BCA assay (Thermo Scientific, Rockford, IL) was performed using a bovine serum albumin standard curve made with 0.5 N NaOH, also heated in the manner described for the samples.

Three separate experiments with duplicate bioreactors for each strain were performed for a total of six replicates each. There was significant batch-to-batch variation for both strains among experimental replicates, with normalized current ranges of 0.06–0.38 µA/µg for Δ*llpR* and 0.19–0.60 µA/µg for MR-1 + *gfp*. However, the ratios of the normalized currents of Δ*llpR* to MR-1 + *gfp* were consistent between batches. Therefore, the final normalized current ratio (0.534 ± 0.077) is an average of pairwise comparisons for averaged replicates with standard error mean. Two separate paired *t*-tests were conducted accounting for each possible pairing of replicates, and the highest resulting *p* value is reported.

#### Theory

Mathematical models are presented in the Supplementary Note.

### Statistics and reproducibility

Statistical tests are defined for each result/experimental method in main text and figure legends. Unless otherwise stated, variance estimates in text are standard error mean.

### Reporting Summary

Further information on research design is available in the Nature Research Reporting Summary linked to this article.

## Supplementary information

Supplementary Information

Descriptions of Additional Supplementary Files

Supplementary Data 1

Reporting Summary

## Data Availability

Authors confirm that all relevant data are included in the article and its supplementary information files. Source data for Figs. 2–4 is provided in Supplementary Data 1. Strains and all other data will be made available upon reasonable request (gralnick@umn.edu).
