# Supplementary material for: Survival of the first rather than the fittest in a Shewanella electrode biofilm
Source: Commun Biol. 2021 May 6;4:536. doi: 10.1038/s42003-021-02040-1 (PMC8102560; doi:10.1038/s42003-021-02040-1)
Supplement: Supplementary file 1 — Supplementary Information [file 42003_2021_2040_MOESM1_ESM.pdf]

Supplementary Information

Survival of the first rather than the fittest in a *Shewanella* electrode biofilm

E.D. Kees, C.E. Levar, S.P. Miller, D.R. Bond, J.A. Gralnick* and A.M. Dean*

*Corresponding authors: gralnick@umn.edu, deanx024@umn.edu

Supplementary Figures 1-4

Supplementary Note

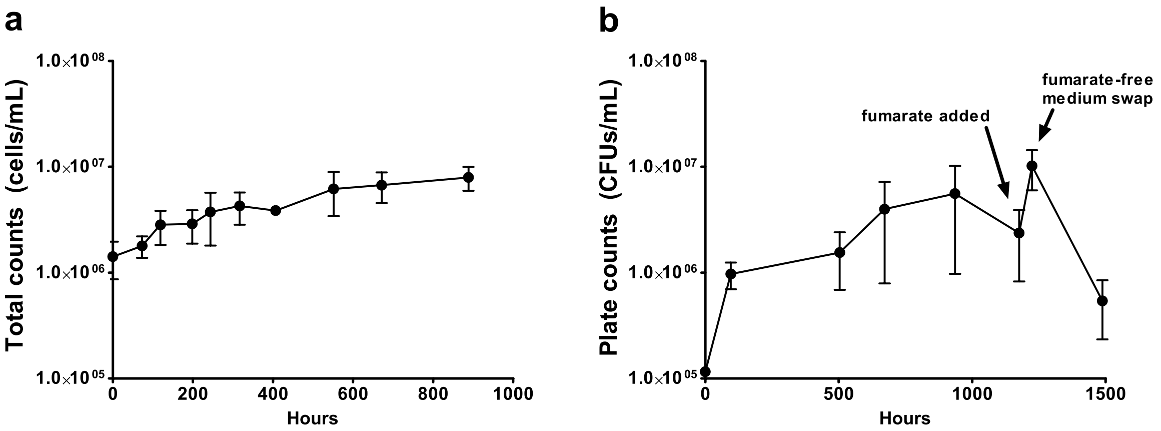

Fig. S1: Total cell counts in biofilm competition experiments. a Non-growing planktonic populations of emigrated anode-grown populations stabilize between 200-400 hours when counting total planktonic cells by flow cytometry across 888 hours (n=2 experimental replicates), or by **b** counting colonies on petri plates across 1488 hours n=3 experimental replicates). An increase in CFUs/mL can be seen upon addition of 5 mM fumarate at 1176 hours and a corresponding decrease in counts can be seen after reactors are washed out with fumarate-free medium. Values are mean and SEM.

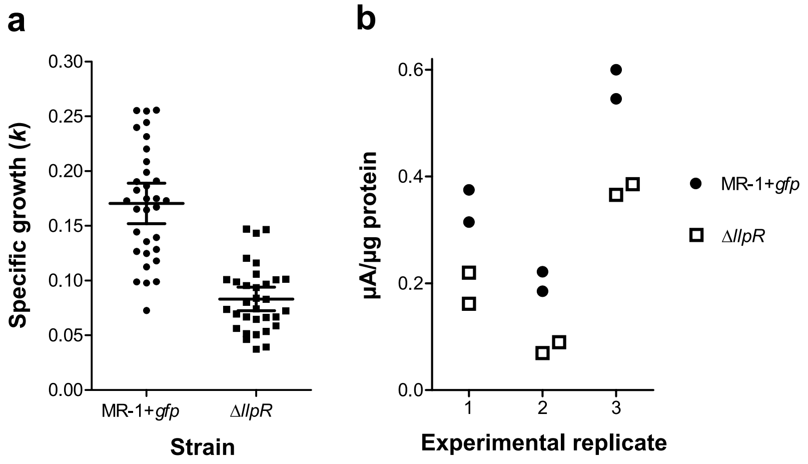

Fig. S2: Specific growth and normalized respiration rates of strains. a Specific growth rates calculated by cells/electrode estimates, divided by total cells removed per hour from bioreactors. See Materials and methods for details. Each data point represents a specific growth rate calculation per subsampled confocal micrograph for each of two time points used to measure medium flow rates and planktonic cell concentrations. Data are representative of two electrodes per strain, two time points and 8 subsampled images per electrode, for n=32 replicates. Estimated growth rates are 0.083 ± 0.005 hr^{-1} and 0.170 ± 0.009 hr^{-1} for Δ*llpR* and MR-1+*gfp* respectively; $p < 0.0001$ by Welch's t-test. Values are mean and SEM. **b** Estimated current to protein ratio (normalized respiration rate) for two replicate bioreactors for each strain across three separate experiments. Values are mean and SEM.

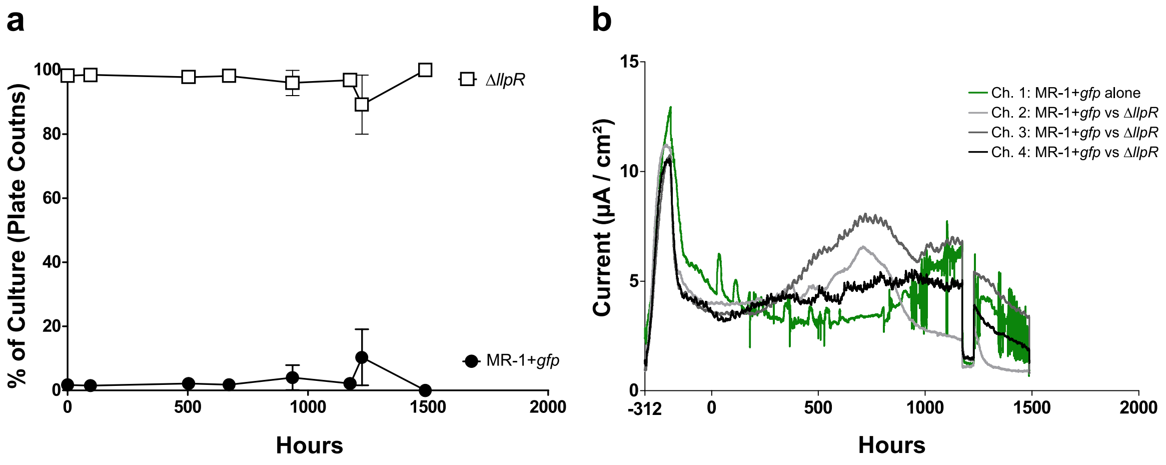

Fig. S3: Relative strain abundance and total biofilm current changes with fumarate spike-in. **a** Percent abundance of competing 2-month old surface-attached biofilms measured by plating planktonic cells. The relative abundances of MR-1+*gfp* (open squares) and Δ*llpR* (black circles) were stable during anodic competition. Strain MR-1+*gfp* began to outcompete strain Δ*llpR* at 1176 hours when 5 mM fumarate was added to the medium. On switching back to fumarate-free medium strain MR-1+*gfp* returned to its original relative abundance by 1500 hours as cells grown on the anodes repopulated the planktonic population washed from the bioreactor (n=3 bioreactors, values are mean and SEM). **b** Chronoamperometry traces from the same experiment show a decrease in the anodic current at 1176 hours as cells preferentially used the added fumarate as a terminal electron acceptor, and corresponding current restoration when the fumarate was removed.

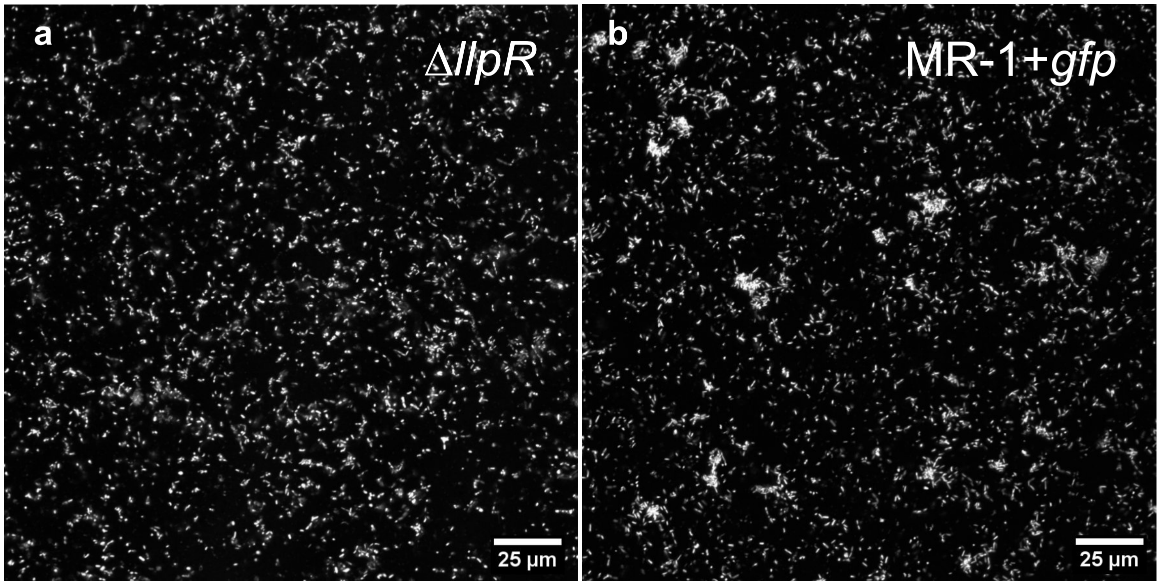

Fig. S4: Representative confocal microscopy maximum intensity projections of monoculture anodic biofilms. Strains Δ*llpR* (**a**) and MR-1+*gfp* (**b**) after 301 hours of electrode surface growth under strict anoxic conditions. For purposes of comparison, each strain was imaged on the same color channel using Syto41 dye.

Supplementary Note

For equation legibility, strain MR-1+*gfp* is referred to as gfp^+ here. Consider two competing strains, densities $N_{\Delta llpR}$ and $N_{gfp}{}^+$, growing exponentially with rates $r_{\Delta llpR}$ and $r_{gfp}{}^+$.

$$\frac{dN_{\Delta llpR}}{dt} = \left(r_{\Delta llpR} - D\right)N_{\Delta llpR} \tag{1}$$

$$\frac{dN_{gfp^+}}{dt} = \left(r_{gfp^+} - D\right)N_{gfp^+} \tag{2}$$

where D is the dilution rate of the bioreactor ($D = 0$ in batch cultures). The densities at time t are found by integration,

$$N_{\Delta llpR.t} = N_{\Delta llpR.0}\, e^{\left(r_{\Delta llpR} - D\right)t} \tag{3}$$

$$N_{gfp^+.t} = N_{gfp^+.0}\, e^{\left(r_{gfp^+} - D\right)t} \tag{4}$$

where $N_{\Delta llpR.0}$ and $N_{gfp+.0}$ are the densities at time zero. The selection coefficient (the difference in the exponential growth rates of the two strains) can be estimated using linear regression as the slope ($s = r_{\Delta llpR} - r_{gfp+}$ of the line of the Log_e ratio of the strain densities plotted against time,

$$Log_e\left[\frac{N_{\Delta llpR.t}}{N_{gfp^+.t}}\right] = Log_e\left[\frac{N_{\Delta llpR.0}}{N_{gfp^+.0}}\right] + st \,. \tag{5}$$

Equation 1 applies to exponential growth in bioreactors, chemostats and batch cultures. Relative growth rate (fitness) is estimated as $r_{\Delta llpR}/r_{gfp+} = 1 + s/r_{gfp+}$.

Now consider two competing populations growing on poised electrodes. Natural selection ceases once the electrodes are completely colonized, as all offspring must now

emigrate. The equilibrium growth rates on the electrodes are zero ($dN_{\Delta llpR.E}/dt = 0$ and $dN_{gfp+.E}/dt = 0$). The growth rates of the planktonic populations are

$$\frac{dN_{\Delta llpR.P}}{dt} = r_{\Delta llpR.E} N^{*}_{\Delta llpR.E} - DN_{\Delta llpR.P} \tag{6}$$

$$\frac{dN_{gfp^{+}.P}}{dt} = r_{gfp^{+}.E} N^{*}_{gfp^{+}.E} - DN_{gfp^{+}.P} \tag{7}$$

where the subscripts *P* and *E* designate the planktonic and electrode phases and the asterisks denote equilibrium population densities. Integrating yields

$$N_{\Delta llpR.P.t} = \frac{r_{\Delta llpR.E} N^{*}_{\Delta llpR.E}}{D} + \left(N_{\Delta llpR.P.0} - \frac{r_{\Delta llpR.E} N^{*}_{\Delta llpR.E}}{D} \right) e^{-Dt} \tag{8}$$

$$N_{gfp^{+}.P.t} = \frac{r_{gfp^{+}.E} N^{*}_{gfp^{+}.E}}{D} + \left(N_{gfp^{+}.P.0} - \frac{r_{gfp^{+}.E} N^{*}_{gfp^{+}.E}}{D} \right) e^{-Dt} \tag{9}$$

The planktonic populations converge to their equilibrium values

$$N^{*}_{\Delta llpR.P} = \frac{r_{\Delta llpR.E}}{D} N^{*}_{\Delta llpR.E} \tag{10}$$

$$N^{*}_{gfp^{+}.P} = \frac{r_{gfp^{+}.E}}{D} N^{*}_{gfp^{+}.E} \tag{11}$$

at a rate determined by the dilution rate, *D*. We see that the equilibrium planktonic population densities (on the left) are proportional to their equilibrium electrode population densities (on the right). Any changes in the latter will be reflected as changes in the former. Counting the planktonic populations allows the electrode populations to be monitored.

On taking the ratio of the planktonic population densities,

$$\frac{N^{*}_{\Delta llpR.P}}{N^{*}_{gfp^{+}.P}} = \left(\frac{r_{\Delta llpR.E}}{r_{gfp^{+}.E}} \right) \frac{N^{*}_{\Delta llpR.E}}{N^{*}_{gfp^{+}.E}}, \tag{12}$$

we see that the ratio of the strains in the planktonic phase equals the ratio of the strains on the electrode times the relative growth rate on the electrode. Natural selection has ceased, both on the electrodes and in the planktonic phase.

This model ignores growth in the planktonic phase, cell death, mutation, and re-colonization of newly exposed anodic surfaces from the planktonic phase. Our data suggest that these processes, if present, are sufficiently weak not to have compromised our conclusions.

Death on electrodes without invasion

We first explore a model in which dead cells on the electrode are not replaced, neither from cells growing on the electrode nor from cells in the planktonic phase. Let δ represent death rates, with subscripts to denote strain and location.

$$\frac{dN_{\Delta llpR.E}}{dt} = -\delta_{\Delta llpR.E} N_{\Delta llpR.E} \tag{13}$$

$$\frac{dN_{gfp^+.E}}{dt} = -\delta_{gfp^+.E} N_{gfp^+.E} \tag{14}$$

$$\frac{dN_{\Delta llpR.P}}{dt} = r_{\Delta llpR.E} N_{\Delta llpR.E} - DN_{\Delta llpR.P} - \delta_{\Delta llpR.P} N_{\Delta llpR.P} \tag{15}$$

$$\frac{dN_{gfp^+.P}}{dt} = r_{gfp^+.E} N_{gfp^+.E} - DN_{gfp^+.P} - \delta_{gfp^+.p} N_{gfp^+.P} \tag{16}$$

Integrating yields,

$$N_{\Delta llpR.E}(t) = N_{\Delta llpR.E}(0) e^{-\delta_{\Delta llpR.E} t} \tag{17}$$

$$N_{gfp^+.E}(t) = N_{gfp^+.E}(0) e^{-\delta_{gfp^+.E} t} \tag{18}$$

$$N_{\Delta IlpR.P}(t) = N_{\Delta IlpR.P}(0)e^{-(D+\delta_{\Delta IlpR.P})t} + \frac{r_{\Delta IlpR.E}N_{\Delta IlpR.E}(0)}{D+\delta_{\Delta IlpR.P}-\delta_{\Delta IlpR.E}}\left(e^{-\delta_{\Delta IlpR.E}t} - e^{-(D+\delta_{\Delta IlpR.P})t}\right) \tag{19}$$

$$N_{gfp^+.P}(t) = N_{gfp^+.P}(0)e^{-(D+\delta_{gfp^+.P})t} + \frac{r_{gfp^+.E}N_{gfp^+.E}(0)}{D+\delta_{gfp^+.P}-\delta_{gfp^+.E}}\left(e^{-\delta_{gfp^+.E}t} - e^{-(D+\delta_{gfp^+.P})t}\right) \tag{20}$$

The dilution rate is far bigger than the death rates ($D \gg \delta$'s) so that, after a short period of adjustment, a steady state is reached characterized by exponential decay in the planktonic populations,

$$N_{\Delta IlpR.P}(t) = \frac{r_{\Delta IlpR.E}N_{\Delta IlpR.E}(0)}{D+\delta_{\Delta IlpR.P}-\delta_{\Delta IlpR.E}}e^{-\delta_{\Delta IlpR.E}t} \tag{21}$$

$$N_{gfp^+.P}(t) = \frac{r_{gfp^+.E}N_{gfp^+.E}(0)}{D+\delta_{gfp^+.P}-\delta_{gfp^+.E}}e^{-\delta_{gfp^+.E}t} \tag{22}$$

Death rates of 0.0005 hr^{-1} would be sufficient to halve the initial planktonic population density over the course of a 1600 *hr* experiment. No decline in the planktonic population was detected. We conclude that alone, death on the electrodes cannot account for our results.

Death on electrodes with replacement by invasion

We now explore a model in which dead cells on the electrode are replaced, either from cells growing on the electrode or from cells in the planktonic phase or both. Let i represent immigration constants, with subscripts to denote strain and location. We assume that immigration constants from the electrode, i_E, are equal and that the immigration rates are proportional to the growth rates of the strains. We assume that immigration constants from the planktonic phase, i_P, are equal (neither strain can metabolize in the absence of a terminal electron acceptor) so that the immigration rates are proportional to the strain densities in the planktonic phase. For computational ease, and without much loss of generality, assume that the death rates, δ_E and δ_P, are the same for both strains. The model becomes,

$$\frac{dN_{\Delta llpR.E}}{dt} = -\delta_E N_{\Delta llpR.E} + \left(i_{\mathrm{E}} r_{\Delta llpR.E} N_{\Delta llpR.E} + i_P N_{\Delta llpR.P}\right) N_a \tag{23}$$

$$\frac{dN_{gfp^+.E}}{dt} = -\delta_E N_{gfp^+.E} + \left(i_E r_{gfp^+.E} N_{gfp^+.E} + i_P N_{gfp^+.P}\right) N_a \tag{24}$$

$$\frac{dN_{\Delta llpR.P}}{dt} = r_{\Delta llpR.E} N_{\Delta llpR.E} - DN_{\Delta llpR.P} - \delta_P N_{\Delta llpR.P} - \left(i_{\mathrm{E}} r_{\Delta llpR.E} N_{\Delta llpR.E} + i_P N_{\Delta llpR.P}\right) N_a \tag{25}$$

$$\frac{dN_{gfp^+.P}}{dt} = r_{gfp^+.E} N_{gfp^+.E} - DN_{gfp^+.P} - \delta_P N_{gfp^+.P} - \left(i_E r_{gfp^+.E} N_{gfp^+.E} + i_P N_{gfp^+.P}\right) N_a \tag{26}$$

where N_a is the open space on the electrode represented in terms of cell numbers.

Assume there is little space available on the electrode ($N_a \approx 0$, confirmed by confocal microscopy, Figure X) so that emigration rates from the planktonic phase onto the electrode are tiny. The planktonic populations will rapidly approach a steady state characterized by $dN_{\Delta llpR.P} / dt \approx 0$ and $dN_{gfp^+.P} / dt \approx 0$. Ignoring emigration to the electrode because the rates are tiny and solving yields,

$$N_{\Delta llpR.P} \approx \frac{r_{\Delta llpR.E}}{D + \delta_P} N_{\Delta llpR.E} \tag{27}$$

$$N_{gfp^+.P} \approx \frac{r_{gfp^+.E}}{D + \delta_P} N_{gfp^+.E} \tag{28}$$

The planktonic population densities are proportional to the electrode population densities. Assume that the changes in space available on the electrode are slow (the assumption of a quasi-steady-state). Then,

$$\frac{dE}{dt} = \frac{dN_{\Delta llpR.E}}{dt} + \frac{dN_{gfp^+.E}}{dt} = \delta_E N_{gfp^+.E} + \delta_E N_{\Delta llpR.E}$$
$$-\left(i_{\mathrm{E}} r_{\Delta llpR.E} N_{\Delta llpR.E} + i_P N_{\Delta llpR.P}\right) N_a - \left(i_E r_{gfp^+.E} N_{gfp^+.E} + i_P N_{gfp^+.P}\right) N_a \approx 0 \tag{29}$$

and so,

$$N_a \approx \frac{\delta_E\left(N_{\Delta IlpR.E}+N_{gfp^+.E}\right)}{i_E\left(r_{\Delta IlpR.E}N_{\Delta IlpR.E}+r_{gfp^+.E}N_{gfp^+.E}\right)+i_P\left(N_{\Delta IlpR.P}+N_{gfp^+.P}\right)}$$

$$\approx \frac{\delta_E\left(N_{\Delta IlpR.E}+N_{gfp^+.E}\right)}{\left(i_E+\frac{i_P}{D+\delta_P}\right)\left(r_{\Delta IlpR.E}N_{\Delta IlpR.E}+r_{gfp^+.E}N_{gfp^+.E}\right)} \tag{30}$$

Substituting the solution for N_a into equations 23 and 24 and rearranging yields

$$\frac{dN_{\Delta IlpR.E}}{dt}=-\frac{dN_{gfp^+.E}}{dt}=\delta_E\left(r_{\Delta IlpR.E}-r_{gfp^+.E}\right)\frac{N_{\Delta IlpR.E}N_{gfp^+.E}}{r_{\Delta IlpR.E}N_{\Delta IlpR.E}+r_{gfp^+.E}N_{gfp^+.E}} \tag{31}$$

The selection coefficient, $\delta_E(r_{\Delta IlpR.E}-r_{gfp^+.E})$, is independent of immigration rates.

For comparison, equations 1 and 2 can be rewritten into a similar form by setting $dN_{\Delta IlpR.P}/dt+dN_{gfp^+.P}/dt=0$, solving for D and substituting. The result is

$$\frac{dN_{\Delta IlpR.P}}{dt}=-\frac{dN_{gfp^+.P}}{dt}=\left(r_{\Delta IlpR.P}-r_{gfp^+.P}\right)\frac{N_{\Delta IlpR.P}N_{gfp^+.P}}{N_{\Delta IlpR.P}+N_{gfp^+.P}} \tag{32}$$

Hence, selection on the electrode is effectively weakened by the factor δ_E, the death rate.
