# Supplementary material for: Survival of the first rather than the fittest in a Shewanella electrode biofilm
Source: Commun Biol. 2021 May 6;4:536. doi: 10.1038/s42003-021-02040-1 (PMC8102560; doi:10.1038/s42003-021-02040-1)
Supplement: Supplementary file 2 — Descriptions of Additional Supplementary Files [file 42003_2021_2040_MOESM2_ESM.pdf]

Descriptions of Additional Supplementary Files

Supplementary Data
Description: Source data for Figures 2-4.
