# Supplementary material for: Survival of the first rather than the fittest in a Shewanella electrode biofilm
Source: Commun Biol. 2021 May 6;4:536. doi: 10.1038/s42003-021-02040-1 (PMC8102560; doi:10.1038/s42003-021-02040-1)
Supplement: Supplementary file 4 — Reporting Summary [file 42003_2021_2040_MOESM4_ESM.pdf]

nature research

Corresponding author(s): Gralnick, JA

Last updated by author(s): Mar 22, 2021

Reporting Summary

Nature Research wishes to improve the reproducibility of the work that we publish. This form provides structure for consistency and transparency in reporting. For further information on Nature Research policies, see our Editorial Policies and the Editorial Policy Checklist.

Statistics

For all statistical analyses, confirm that the following items are present in the figure legend, table legend, main text, or Methods section.

n/a	Confirmed	
☐	☒	The exact sample size (n) for each experimental group/condition, given as a discrete number and unit of measurement
☐	☒	A statement on whether measurements were taken from distinct samples or whether the same sample was measured repeatedly
☐	☒	The statistical test(s) used AND whether they are one- or two-sided<br>*Only common tests should be described solely by name; describe more complex techniques in the Methods section.*
☒	☐	A description of all covariates tested
☐	☒	A description of any assumptions or corrections, such as tests of normality and adjustment for multiple comparisons
☐	☒	A full description of the statistical parameters including central tendency (e.g. means) or other basic estimates (e.g. regression coefficient) AND variation (e.g. standard deviation) or associated estimates of uncertainty (e.g. confidence intervals)
☒	☐	For null hypothesis testing, the test statistic (e.g. F, t, r) with confidence intervals, effect sizes, degrees of freedom and P value noted<br>*Give P values as exact values whenever suitable.*
☒	☐	For Bayesian analysis, information on the choice of priors and Markov chain Monte Carlo settings
☒	☐	For hierarchical and complex designs, identification of the appropriate level for tests and full reporting of outcomes
☒	☐	Estimates of effect sizes (e.g. Cohen's d, Pearson's r), indicating how they were calculated

Our web collection on statistics for biologists contains articles on many of the points above.

Software and code

Policy information about availability of computer code

Data collection	*Provide a description of all commercial, open source and custom code used to collect the data in this study, specifying the version used OR state that no software was used.*
Data analysis	*Provide a description of all commercial, open source and custom code used to analyse the data in this study, specifying the version used OR state that no software was used.*

For manuscripts utilizing custom algorithms or software that are central to the research but not yet described in published literature, software must be made available to editors and reviewers. We strongly encourage code deposition in a community repository (e.g. GitHub). See the Nature Research guidelines for submitting code & software for further information.

Data

Policy information about availability of data

All manuscripts must include a data availability statement. This statement should provide the following information, where applicable:

- Accession codes, unique identifiers, or web links for publicly available datasets
- A list of figures that have associated raw data
- A description of any restrictions on data availability

The authors declare that all data supporting the findings of this study are available within the paper and its supplementary information files.

[illegible]

[illegible]

- [x] [illegible]
- [ ] [illegible]
- [ ] [illegible]

[illegible]

[illegible]

[illegible]

[illegible]	[illegible]
[illegible]	[illegible]
[illegible]	[illegible]
[illegible]	[illegible]
[illegible]	[illegible]

[illegible]

[illegible]

[illegible]

[illegible]	[illegible]
[x] [ ]	[illegible]
[x] [ ]	[illegible]
[x] [ ]	[illegible]
[x] [ ]	[illegible]
[x] [ ]	[illegible]
[x] [ ]	[illegible]
[x] [ ]	[illegible]

[illegible]

[illegible]	[illegible]
[x] [ ]	[illegible]
[ ] [x]	[illegible]
[x] [ ]	[illegible]

[illegible]

[illegible]

[illegible]

- [ ] [illegible]
- [ ] [illegible]
- [ ] [illegible]
- [ ] [illegible]

[illegible]

[illegible]	[illegible]
[illegible]	[illegible]
[illegible]	[illegible]
[illegible]	[illegible]
[illegible]	[illegible]

[illegible]

☐ Tick this box to confirm that a figure exemplifying the gating strategy is provided in the Supplementary Information.
